# Impact of Early Dressing Removal on Tunneled Central Venous Catheters: A Piloting Study

**DOI:** 10.31557/APJCP.2019.20.9.2693

**Published:** 2019

**Authors:** Ghada Ammar, Ezzaldeen Almashaikh, Ahmad Ibdah, Waleed Shajrawi, Safwat Awawdeh, Ayoub AL Mousa, Belal AL-Blowi, Moh’d Baseem Hamdan, Amani Al Eleiwah, Wala Al Jabali, Hussien Hussien, Abdelrahman Salameh, Mohammad Alkharabsheh

**Affiliations:** *King Hussein Cancer Center, Jordan. *

**Keywords:** Tunnel Central Venous Catheter, no-dressing, well-healed exit site

## Abstract

**Background:**

Central Venous Catheters (CVC) are linked with Catheter-related bloodstream infections (CLABSI) or exit-site infections. Dressings may reduce the rate of infection, but they are uncomfortable, do not eliminate the risk of infection, and in some cases become the cause of infection.

**Aim:**

This study evaluates the impact of early CVC dressing removal on CLABSI, exit-site infections, and patient quality of life in an oncology setting.

**Method:**

A quasi-experimental pilot study was conducted over 15 months at a specialized oncology center. Sixteen patients were divided into control (n=8) and experimental (n=8) groups. The control group received the standard protocol of applying CVC dressings, while the experimental group received a “no-dressing” protocol.

**Results:**

There was no statistical significance in the infection rate between the two groups (p=1.0). Two cases developed CLABSIs, one in each group. One patient from the experimental group developed an exit-site infection as well. Patients in the experimental group reported high satisfaction and an improved quality of life.

**Conclusions:**

Applying a no-dressing protocol to a well-healed exit site CVC showed encouraging results in terms of exit-site and bloodstream infections. That is to say; it did not predispose patients to increased risk of infections. Furthermore, patients with no dressing protocol feel more comfortable in their life.

## Introduction

A Central Venous Catheter (CVC) is an intravascular catheter close to the heart. Most medical teams consider early CVC as a way for infusion, withdrawal of blood or hemodynamic monitoring (Murphy et al., 2009). With oncology patients, CVC facilitates the administration of chemotherapy and other medications over prolonged periods of time, in some cases, lasting years, preserving patients’ peripheral veins and improving their quality of life (Marschall et al., 2014; Parienti, et al., 2015 Schiffer et al., 2013). 

However, CVC is linked with immediate or delayed complications, which are further divided into three categories; mechanical, embolic, and infectious complications (Smith and Nolan, 2013).

Infections associated with CVC are one of the leading issues influencing patients’ treatment plans, as they cause an increase in the intervals of treatment and can be a threat to life if not determined immediately (O’Grady et al., 2011). Central line–associated bloodstream infections (CLABSIs) are defined as bacteremia in a patient with an intravascular catheter accompanied by clinical manifestations of the infection (i.e., fever, chills, and/or hypotension). Bloodstream infections are linked with CVC if the line was in use during the 48-hour period before the progress of the bloodstream infection (O’Grady et al., 2011).

Exit-site infections can be defined as a discharge from the catheter exit-site yielding a microorganism with or without a concomitant bloodstream infection. The infection may be accompanied by tenderness within 2 cm of the catheter exit-site; and with or without any other signs and symptoms of infection, such as fever (Murphy et al., 2009).

**Table 1:** Patients Demographic Characteristics

		Total	Control group	Experimental group	p-value
Gender	Female	3 (18.75%)	2 (66.67%)	1 (33.33%)	1
	Male	13 (81.25%)	6 (46.15%)	7 (53.85%)	
Participants (n = 16 )	Study finished while patients had the line	8 (50%)	5 (62.5%)	3 (37.5%)	0.78
	Patients whose line was removed because of end of treatment	5 (31.25%)	2 (25%)	3 (37.5%)	
	Patients who left the study because of exit site infection	1 (6.25%)	0 (0.0%)	1 (12.5%)	
	Patients who left the study because of blood stream infection	2 (12.5%)	1 (12.5%)	1 (12.5%)	
Total		16 (100%)	8 (100%)	8 (100%)	

**Table 2:** Patients average Age

		Average	Control group	Experimental group
Age	Female	3 (23.6 years)	2 (23 years)	1 (25 years )
	Male	13 (27 years )	6 (38.5 years)	7 (32 years )

**Table 3:** Patient's Diagnosis

Diagnosis (n = 16 )	Hodgkin's Lymphoma (6)
	Non-Hodgkin's Lymphoma (1)
	MDS/Myeloid Sarcoma (1)
	AML (2)
	ALL (3)
	Lymphoma (1)
	Other (2)

CVC dressing is shown to be effective in preventing CLABSIs, as it provides a protective barrier, preventing the migration of skin organisms at the insertion site into the catheter tunnel and the subsequent colonization of the catheter tip. It also prevents direct catheter contamination through contact with hands and other materials (O’Grady et al., 2011).

Dressing is usually applied on the exit-site of the CVC after cleaning the site by an antiseptic, this cleansing technique for catheter care is the most important factor in preventing infection. A meta-analysis study reported that some interventions, such as maximal sterile precautions during insertion, skin antisepsis, securement devices (dressing) and antimicrobial catheter coatings can reduce CLABSIs (Han et al., 2010). 

Furthermore, a 2015 study reported that decreasing the incidences of CLABSIs and preventing CVC failure have a significant impact on patient morbidity and mortality. However, there is no agreement on the best dressing or securement type to use with CVCs even after two decades of research on the topic (Ullman Amanda et al., 2015).

According to a guideline that was published in 2011 for the prevention of intravascular catheter–related infections, it was stated that the importance of an exit-site dressing on CVCs was an unresolved issue (O’Grady et al., 2011). The no-dressing protocol was considered into the guidelines for the prevention of intravascular catheter-related infections. Studies as early as 2004 show that keeping the site without a dressing may reduce catheter-related blood stream infections (Olson et al., 2004), especially since the dressing itself may become a source of infection.

A pilot study investigated a protocol of applying no dressing over hemodialysis catheter exit-sites compared the outcomes of two groups of patients (a shower group versus a non-shower group) within six months across multiple sites. The results showed that there was no significant difference between the two groups either exit-site infection rates, tunnel infection rates, or CLABSI (Evans et al., 2014).

Moreover, a quality improvement study stated that the “shower and no-dressing” technique appears to be safe as CVC care option, with improved quality of life, cost-effectiveness, and decreased infection rates. Also, after 1,380 catheter days (n = 119), infection rates were 0.31 events per 1,000 catheter days among those who performed the shower and no-dressing technique (Lawrence and Wilson, 2014). This 2014 study shows that patients are given new options in managing their lives, by giving them freedom from the dressing and independence in their daily activities. The no-dressing approach may also help medical teams in decreasing infections, especially with patients who have skin problems or high risk of infection.

Our study aims to evaluate the effect of the dressing versus early removal of the dressing on well-healed, tunneled, cuffed CVCs on infection and quality of patients’ lives.

## Materials and Methods

*Settings*

The research was conducted at the King Hussein Cancer Center (KHCC). KHCC is a specialized oncology center and the only specialized hospital implementing comprehensive cancer care in Jordan. KHCC receives patients with cancer from across Jordan and neighboring countries. It is a certified center with high technological and well-equipped facilities, and a capacity of 352 beds (KHCC, 2019).

The Venous Access Device (VAD) team is one of the specialized nurses’ teams working at the center. Their main tasks are providing daily nursing care for all patients who have CVCs inside the center; changing the dressing, blood extracting, and caring for CVC complications. VAD practice is based on best practices and international guidelines (KHCC, 2019).

*Design*

A quasi-experimental pilot study design was conducted at KHCC from November 2015 to July 2016. Patients who met the inclusion criteria were divided into two groups; a control group and an experimental group. The participants were divided into groups to ensure that the experimental and control groups are comparable in all regards (distribution of potential confounding factors).

*Sample*

The inclusion criteria were adult (18 years or older) patients of varied diagnoses, at the day of long-term tunneled CVC (Hickman) insertion. Exclusion criteria included patients who had a history of another primary cancer, community acquired infections, severe psychiatric illness or a bad co-morbid condition and other chronic illnesses like hypertension or diabetes mellitus that can interfere with CVCs during the study. The sample was divided between a control and an experimental group (n=8 in each group). The control group used Chlorhexidine Gluconate (CHG) dressings, while the experimental group did not use a dressing see Figure 1.

*Ethical Consideration*

The ethical approval to conduct this study was obtained from the institutional review board (number: 14 KHCC 57) at KHCC on Aug. 6, 2015”. Participants were ensured that their participation in the study is voluntary. The aim of the study was described clearly to the participants, and they were informed that they could withdraw from the study at any time. Confidentiality and anonymity of information were guaranteed. All participants signed the informed consent prior to participation in the study. The consent form for each participant was allocated with an identification number for more confidentiality. The collected data were saved in a secured folder cabinet.

*Data collection*

Once we received permission from the patients who fulfilled the inclusion criteria, the study procedure was explained, including CVCs and the need for them, their risks and benefits, and how to use them at regular and emergency hospital visits. The patients were also informed that there was no penalty if they refused to participate. Those who agreed to participate signed an informed consent. The participants were consulted whether they prefer to be assigned to the control or experimental groups. 

At the day of insertion, the patients of both the control and the experimental groups had a full assessment for clinical status and treatment plan. The patients’ primary doctors were consulted, and their permission was requested for patients to enroll in this study. After insertion, the patients were assessed for any abnormalities at the exit-site (e.g. pain, bleeding, tenderness), then chest x-rays were done to ensure proper catheter position. 

Patients had gauze dressing for the first 48 hours after CVC insertion, then a CHG dressing was used if there was no blood oozing for the first three weeks. The Center uses transparent dressing for all patients unless the patient presents with any skin abnormalities (redness, tenderness, hematoma, or any discharge). 

If the patient had been admitted to the Center, the VAD team followed up. In the case of out-patients, they were asked to return after two days, so a VAD nurse could assess whether to switch to a transparent dressing or to keep using a gauze dressing on case by case basis. If the patient switched to a transparent dressing, it was changed every 7 days for all patients and when needed. 

The control group continued to use the dressing after those initial three weeks and came for weekly visits and when needed. Patients were trained for the shower routine at home, by covering the line with special bags and tape (provided by the Center) during the shower. Moreover, the catheters were to be cleaned with chlorhexidine swabs by anchoring the catheter at the exit-site, and gently swabbing from the proximal to the distal end. The Hickman line to be looped in a circle away from the insertion site.

As for the experimental group, after the initial three weeks, the dressing was removed upon patient approval and after the assessment of a co-investigator physician (surgeon). The patients were instructed to visit every three to five days in the first month, then weekly after that (and when needed), to assess exit-site healing and check the CVC, this entailed a normal skin color, no redness, no tenderness, no hematoma, and no discharge. A shower protocol using 4% CHG shampoo is detailed in the Appendix.

Six months after the enrollment in the study, the patients in the experimental group were given the option to return to the standard dressing practice. 

During the study, if any patient dropped out of the study for any reason, they were offered the standard level of care at the Center. 

The authors also explained to the patients in both groups that their visits for exit-site assessments did not substitute for their regular visits to other services. Therefore, their visits to other services offered by the Center were not recorded in our study.

After the study, a semi-structured interview was conducted with the patients in the experimental group in a private room. The interview was around 10 minutes long, where patients were asked how they felt about their life without putting a dressing, their quality of life, and why they thought that the no-dressing protocol was suitable for them. All their feedback was recorded.

*Data Analysis*

The collected data was entered and analyzed using Statistical Analysis System (SAS version 9.4). Descriptive and inferential statistics were used to report sample variables at a significance level of p=.05. Descriptive analysis of the patients’ information was conducted. Gender and causes of leaving the study are presented as frequencies and percentages. In general, differences in proportions were tested with Fisher exact test.

## Results

The minimum required total sample size for a two-arm explorative pilot trial following some currently proposed approximation guidelines is around 20 participants (Whitehead et al., 2016). However, in our sample were 16 patients (8 in each group). Most (n=13; 81.25%) were male. 

Eight (50%) participants remained with the CVC until the end of the study; five (62.5%) of the control group, and three (37.5%) of the experimental group. Five (31.25%) participants completed their treatment and removed the CVCs thus left during the course of the study; 2 (25%) of the control group, and 3 (37.5%) of the experimental group. 

After being assigned to groups, the groups were assessed for any significant differences. There were no significant differences in CLABSI rates between the two groups (p=1.0). Two cases from the experimental group developed infections; one with a positive blood culture or bloodstream infection (BSI), and another developed an exit-site infection. While in the control group, only one patient developed a BSI.

The interview with the experimental group patients revealed that their quality of life was enhanced without increasing their rate of infection. Only four patients were directly asked “*why do you think that the no-dressing protocol was suitable for you?*”, their answers were:

• “*My life became easier. Bathing is easier without restrictions, and I became more independent.*”

• “*An excellent and an interesting idea.*” “*My life became easier, bathing too.*”

• “It gives me more freedom in my personal life.” “I feel like a normal person.”

• “*It is good for me, and I hope it will help in the future with other patients*”.

After six months, once given the option, none of the patients in the experimental group requested to go back to the dressing protocol.

## Discussion

One of the complications of chemotherapy is CVC infections, and CVC infections in turn may delay treatment times and affect the patients’ quality of life. Medical centers and researchers aim to improve CVC care by finding best practices to protect patients’ lives and decrease infections (Coady, et al., 2015; Patel et al., 2014).

Using a dressing protocol for CVCs after applying a proper antiseptic has been proposed. However, the dressing protocol requires more frequent hospital visits, as follow up is needed for changing and managing the issues associated with it. Moreover, in the summer, some patients need to come to the hospital on daily basis because of excessive sweating, which causes the dressing to become loose. Patients have no choice but to adjust to CVC dressing care. Dressings should be changed when they become damp, loose, soiled, non-occlusive, or non-adherent, and only trained staff should change catheter dressings. Some patients have developed skin allergy or contact dermatitis related with sweat, especially with the use of the CHG dressing. All these factors would affect the CVC exit-site and would lead to severe itching, infection, severe abrasion, and septicemia in immunocompromised patients with cancer. The use of dressings does not eliminate the problem of infection. In some cases, it may even be the cause of infection.

Some physicians decide to remove the dressing, as contact dermatitis disrupts patients’ lives. Fortunately, the patient’s status improves after removing the dressing. The hypothesis suggests that eliminating CVC exit-site dressing may reduce CLABSIs. 

Only four studies addressed the effect of using a no-dressing protocol on the catheter exit-site in relation with CLABSIs, tunneled infections, and exit-site infections (Evans et al., 2014; Lawrence and Wilson, 2014; Steen et al., 2019; Webster et al., 2017). The studies reported that removing the dressing had a positive impact on the prevention of exit-site infections. These conclusions support the results of our study which showed that the removal of the dressing along with the prescribed shower regimen have a great impact on the management of these patients. 

Moreover, the results showed that only two cases developed CLABSIs, one in each group, and only one case developed an exit-site infection in the (experimental group). This can be explained by participants being oncology patients; therefore, they were immunocompromised during the treatment course.

The participants in the experimental group reported being satisfied with the no-dressing protocol, in line with the results reported in the literature (Lawrence and Wilson, 2014; Coady, et al., 2015; Patel et al., 2014).

Despite the minimum required sample size of 20, we faced a low participation rate. Reasons stated this as a quasi-experiment, also, some doctors were worried about infection, so they did not consent to their patients being enrolled in the study. Females were less likely to accept, as they worried about their lifestyle and personal lives.

*Conclusions and Recommendations*

Dressing removal in patients with tunneled CVCs has a positive effect on their life, patients can bathe without fear of losing the dressing, they feel free without the dressing. Removal of the dressing does not have a significant difference in rate of infection between the control and experimental groups.

The influence on quality-of-life indicators, self-efficacy, and patient agreement is beneficial to lead the practice. Our study proved that the “*shower and no-dressing*” method could have more benefits over our standard practice in a way that does not disturb the patient with a CVC, nor elevates the rates of infection.

A larger scale study is needed to generalize the results, involving multiple centers. Further randomized, controlled clinical trials are recommended to evaluate the safety outline of this technique. 

## Appendix

Participants were asked to follow a shower routine as follows:

1. Gather all that you need before entering the shower.

2. Make sure the catheter caps are secure before showering.

3. In the shower, wash and rinse your face, hair first, then wash the remainder of your body.

4. Wash the catheter site last.

Washing the catheter site (for this procedure use the chlorhexidine bar that was provided):

1. First, once more wash your hands with soap and water.

2. Gently wash the skin around the catheter with soap with your clean hands.

3. Rinse the area well with copious amount of water and do not wash this area again.

4. Once out of the shower, gently pat the skin around the catheter with a clean gauze dressing.

5. Dry the rest of the body with a towel.

6. Open the alcohol prep pad with the provided 2% chlorhexidine. Scrub the skin around the catheter from side to side. Turn the pad over and scrub again this time in an up-down motion

7. Pad the catheter tips with gauze or tape the catheter to your skin per your preference.

8. {Females} be sure to keep the catheter secure by wearing a bra and tucking the ends into the bra.

9. Wear a snug t-shirt to help prevent tugging on the catheter.

Patients were allowed to shower at their own desired frequency but at least two times weekly.
